# Gender, Culture, and Sex-Typed Cognitive Abilities

**DOI:** 10.1371/journal.pone.0039904

**Published:** 2012-07-10

**Authors:** David Reilly

**Affiliations:** School of Applied Psychology, Griffith University, Southport, Queensland, Australia; University Of São Paulo, Brazil

## Abstract

Although gender differences in cognitive abilities are frequently reported, the magnitude of these differences and whether they hold practical significance in the educational outcomes of boys and girls is highly debated. Furthermore, when gender gaps in reading, mathematics and science literacy are reported they are often attributed to innate, biological differences rather than social and cultural factors. Cross-cultural evidence may contribute to this debate, and this study reports national gender differences in reading, mathematics and science literacy from 65 nations participating in the 2009 round of the Programme for International Student Assessment (PISA). Consistently across all nations, girls outperform boys in reading literacy, *d* = −.44. Boys outperform girls in mathematics in the USA, *d = *.22 and across OECD nations, *d* = .13. For science literacy, while the USA showed the largest gender difference across all OECD nations, *d = *.14, gender differences across OECD nations were non-significant, and a small female advantage was found for non-OECD nations, *d* = −.09. Across all three domains, these differences were more pronounced at both tails of the distribution for low- and high-achievers. Considerable cross-cultural variability was also observed, and national gender differences were correlated with gender equity measures, economic prosperity, and Hofstede’s cultural dimension of *power distance*. Educational and societal implications of such gender gaps are addressed, as well as the mechanisms by which gender differences in cognitive abilities are culturally mediated.

## Introduction

Rightly or wrongly, the topic of gender differences in cognitive abilities appears perennial, holding curiosity not only for social scientists but also for the general public and media [1]–[4]. Intelligence is multifaceted [5]–[10], and comprises a range of culturally-valued cognitive abilities. While there is almost unanimous consensus that men and women do not differ in general intelligence [11]–[14], there are several domains where either males or females as a group may show an advantage, such as *visuospatial* [15]–[16] and *verbal* abilities [17]–[18] respectively. However, gender differences in *quantitative abilities* [19], such as science and mathematics, remain contentious. Researchers are divided between arguing for small but still influential differences in quantitative reasoning [9]–[11], and claiming that any observed differences in maths are so small, in fact, that they can be categorised as ‘trivial’ [12]–[14].

A key limitation of research in this area is that it is largely US-centric, and does not speak to gender differences between males and females raised under different social and educational environments in other cultures. Additional lines of evidence are required, and one such source is international testing of students. Secondly, research primarily focuses on mean gender differences, and fails to address gender differences in the tails of distributions which Hyde, et al. [20] argues may forecast the underrepresentation of women in the science, technology, engineering and mathematics (STEM) related professions.

To this aim, I present findings from the 2009 OECD Programme for International Student Assessment (PISA), which to my knowledge has not yet been widely discussed in psychology journals. This information provides a snapshot of current gender differences and similarities in reading, mathematics and science across 65 nations. It also highlights the wide degree of cultural variation between nations, and examines the role that social and environmental factors play in the development of gender differences. Before reviewing the PISA findings, I will briefly discuss the advantages that national and cross-national testing have to offer the debate on the nature of gender differences in cognitive abilities.

### Advantages of Nationally-representative Samples for Assessing Gender Differences

Large national and international samples can provide a ‘yardstick’ estimate of gender differences within a given region, at a given point in time. By drawing from a broad population of students, national and international testing provide us with stronger evidence for gender similarities or differences than could be found from smaller, more selective samples. It is common practice for gender difference studies to use convenience samples drawn from psychology student subject pools [21], as well as from groups of high performing students such as gifted and talented programmes [22] – conclusions drawn from such samples may not be generalizable to wider populations. There is evidence to suggest that the performance of males is more widely distributed, with a greater numbers of high and low achievers [23]. This has been termed the *greater male variability hypothesis* [10], [15]–[16], and presents a problem for researchers recruiting from only high achievers – even though mean differences between males and females may be equal, if the distribution of male scores is wider than females, males will be overrepresented as high-achievers in a selective sample. This may lead to the erroneous conclusion that gender differences exist in the population of males and females.

**Figure 1:**
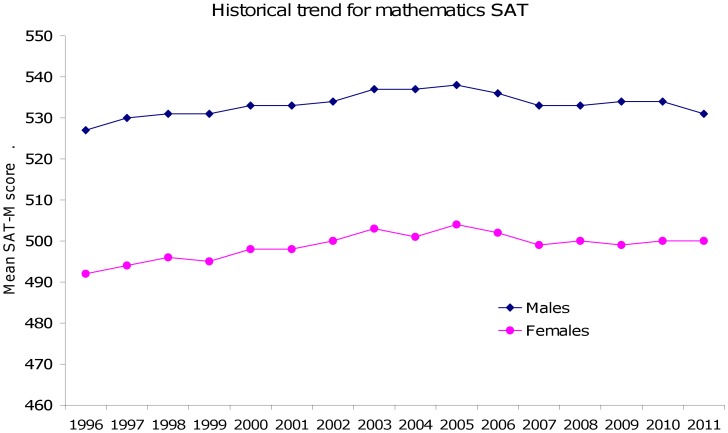
Gender differences in SAT-M performance. On average, boys score higher than girls on the SAT-M exam (approximately one third of a standard deviation). The pattern of scores is consistent across years and does not appear to be diminishing, contrary to other lines of evidence that show gender differences in mathematics are small [51].

A good example of this in practice comes in the form of the Scholastic Assessment Test (SAT) used for assessing suitability of students for college entry within the United States. Males consistently outperform females on the mathematical component [22], [24]–[25]. Gender differences in SAT-M are extremely robust across decades, see Figure 1. On the basis of this evidence alone, one might erroneously conclude that the gender gap in mathematics is pervasive unless consideration is given to the demographics of the sample. Students considering college admission are motivated to undertake the SAT, and this is largely a self-selected sample that may differ on important characteristics such as socioeconomic status, and general ability level. Additionally many more girls sit the SAT than boys [24], [26], reflecting the higher admission rate of women in college [27]. Thus the sample of males is more selective, while the sample of females is more general. One cannot rule out the possibility that the male sample includes a greater proportion of high achieving students and that the female sample may have included students of more mediocre mathematical ability, lowering mean performance.

This does not mean, necessarily, that one should discount any finding of gender differences in the SAT-M as being invalid. Data from the SAT may be extremely useful in estimating gender differences in the population of students considering further education. This is a very narrow, quite specific theoretical question. But such findings cannot be easily generalised to the *general* population, which is what researchers and laypersons alike would seek to test.

Another source of information on gender differences comes from experimental research carried out in the laboratory, under tightly controlled conditions. Equal numbers of males and females can be recruited using random selection. When large samples are randomly drawn from the general population, the scores of both high and low achievers are included in measurements of gender differences. Such studies are time-consuming and expensive to conduct, however. More commonly, gender difference studies use much smaller convenience samples, such as a subject pool of college students which also introduces the problem of selection bias [21]. College subject pools differ from the general population across many different characteristics [28], such as socioeconomic status, general intelligence, and prior educational experiences. Since the scores of males are more variable [12], [18]–[19], a convenience sample that draws from only the upper-tail of ability will be skewed with a greater frequency of high performing males than females, thus exaggerating any gender difference that is found.

Additionally, many cognitive abilities show an interaction between gender and socioeconomic status [1], [25]–[28]. Studies that selectively recruit from college subject pools in medium- to high- socioeconomic status regions would therefore be more likely to find gender differences than those recruiting from lower socioeconomic regions, as there will be greater differentiation between high and low ability levels. Likewise, samples drawing from a college pool may find greater gender differences than if they were recruited from a high school sample, or from the general population. Potentially, this could give a distorted picture of actual gender gaps when generalising from these selective samples to the wider population of males and females.

Large national samples allow researchers to investigate objectively the existence and magnitude of gender differences or similarities. We can be more confident that any observed differences are reflective of what we would find in the general population of boys and girls, and are not simply due to sampling bias. As additional waves of testing are conducted using similar measurement instruments, we can also begin to track any changes over time. It allows us to evaluate efforts aimed at reducing gender differences, and to see areas where further progress must be made. Such data may also be of benefit to policy makers and educational institutions in advocating for educational change, and in support of programs aimed at addressing inequalities.

### Gender Differences in Mathematics and Science within the United States

For the United States, one such program is the National Assessment of Educational Progress (NAEP), a federal assessment of educational achievement. The NAEP is conducted for all states within the United States and since participation is both comprehensive and not self-selected, is ideally suited to answering the question of whether males and females differ in mathematical ability (a type of quantitative reasoning). Hyde [20] and colleagues examined gender differences between boys and girls in mathematics from grades 2 through 11, drawing on a sample of students from ten states which amounted to a sample of over seven million students. Hyde, et al. [20] reported an effect size for gender differences in each grade that approached zero, and categorised differences between males and females as “trivial” [29].

While this evidence seems quite compelling, one must be cautious about generalising the conclusion of ‘no difference’ in maths performance on the NAEP to maths performance in *all* areas of mathematics. As Hyde and Mertz [29] acknowledge, the test content of the NAEP does not include complex test items, making it impossible to investigate gender differences in this area. Complex and novel mathematical problem-solving is a prerequisite skill for success in many academic areas but most particularly in STEM-related fields. With increased affordability and access to calculators and computers, basic computation skills have become less important than the ability to understand complex problems and find strategies to solve them. A comprehensive meta-analysis conducted by Hyde, Fennema and Lamon [30] found small to medium sized differences in complex problem solving favoring males (*d* = .29). Assessment that includes these types of mathematical problems, therefore, should presumably show larger gender differences and might not necessarily support the gender similarities hypothesis. Evidence from the NAEP may exhibit a ceiling effect, as test content hasn’t adequately provided the opportunity for differentiation between high and low ability levels in complex reasoning. This would make the distribution of scores largely homogenous, preventing us from adequately testing the gender differences/gender similarities hypothesis.

### International Sampling of Science and Mathematical Ability

Another source of evidence for evaluating claims of gender differences comes from international testing of students’ educational attainments as part of the OECD’s Programme for International Assessment (PISA). Beginning in 2000 and conducted every three years, participating nations assess the educational attainment of students using a standardized exam that allows their performance to be compared globally. PISA aims to assess the educational progress of students as they reach the end of compulsory education, at age 15, across three skill areas: these being reading literacy, mathematical literacy, and science literacy. Samples are stratified random probability samples, selected from a range of public and private institutions across geographical regions, and weighted so as to be nationally representative [31]. This overcomes the selection-bias of tests such as the SAT-M [24], [26], as well as providing a more valid assessment of the general population of boys and girls at that age than could be found in college-bound students.

Additionally, the test content of PISA is somewhat different to that of other national testing assessments, such as the NAEP. PISA assesses both knowledge and problem-solving skills, reflecting the type of real-world content and skills required to be an informed and capable information consumer and citizen. It assesses a student’s reading, mathematical and scientific literacy, their ability to solve problems and to apply their knowledge and skills across each of these three domains. This is in contrast to tests that require primarily memory of learned material from the curriculum, allowing for greater differentiation between high and low ability levels. As such, it taps higher level cognitive skills than may be found in testing schemes like NAEP, which Hyde and colleagues have reported show small or trivial gender differences in science and mathematics [20]. The test content is sufficiently demanding that only 1.9% of US students are classified as attaining the highest proficiency level in mathematics, and only 1.3% of US students in science. While this makes it ideal for testing for gender differences or similarities within a given country such as the US, it also affords the opportunity to study them cross-culturally.

### Cross-national Variation in Cognitive Abilities

Cross-national variation in the magnitude of gender differences can provide useful information about the environmental conditions that foster, or inhibit, gender differences in domains such as mathematics. While gender differences in mathematics are frequently found at a national level, they are not found universally across *all* nations [32]. Social roles for women vary greatly from culture to culture, with some cultures promoting higher standards of gender equality and access to education than others [33]. Even those nations that have progressive attitudes towards women may still have strongly-held cultural stereotypes that narrowly constrain them [34]–[38]. Cultural stereotypes that girls and women are less able than boys and men in mathematics and science still endure [39]–[40], and these stereotypes have damaging consequences for the self-efficacy of young girls [41].

Cross-cultural comparisons of the performance of males and females might help answer some theoretical questions about the *origins* of any observed gender differences. When we see consistent gender differences across many or all nations, and when they are large enough in magnitude to have a practical impact on the educational and occupational aspirations of boys and girls, then we might reasonably conclude some systematic process is responsible – be this biological or institutional. When we see changes in the *magnitude* and the *direction* of gender differences, as is the case for science performance reported below, then we might reasonably conclude that either cultural or environmental influences are strong moderators in the development of cognitive ability - gender differences are not an inevitable consequence of biology. Finally, if we were to see more *similarities* than differences in the performance of boys and girls, then this would also be useful information for shaping public policy and educational practices such as continuing support for coeducation [42].

A number of previous studies have examined the size of gender differences in cognitive abilities cross-culturally in an attempt to shed light on the underlying causes of such variation. Baker and Jones [43] reported strong correlations between measures of gender equity (such as percentage of females in higher education and the occupational status of women in society) and gender differences in mathematics. Gender differences in mathematics were smaller in more gender-equal nations than in less-equal nations. Though the precise mechanism by which this occurs is unclear, these findings have been replicated by a number of researchers [31]–[32], [43]. This suggests that two factors influencing the cognitive abilities of women are the gender stereotypes that a culture holds, and the gender-roles for women in a society [29], [32]. This has been referred to in the literature as the *gender stratification* hypothesis [33], [43], which argues that gender differences are more pronounced when the roles of men and women are tightly controlled into separate spheres and duties [35], [37], [44]–[45].

Mathematics is not the only cognitive domain where we see an influence of gender-equality and gender stereotypes on cognitive performance. The female advantage in reading and language, while universal, also differs in magnitude between nations. Guiso, et al. [32] examined data from the PISA 2003 round of testing, replicating the finding of Baker and Jones for mathematics as well as finding an association between gender equity and the gender gap in reading. Although this might be expected given that correlations between mathematics performance and reading overlap, the direction of the association differed. Instead of finding reduced gender differences in reading for countries fostering greater gender-equality, the gender gap between boys and girls actually *increased*. One possibility for this seemingly paradoxical finding is that whatever natural advantage girls may have for reading is suppressed in more restrictive countries, but that under favorable conditions is allowed to flourish to its full potential. However, further replication of these findings with subsequent waves of testing is required to determine whether this association is stable across time.

### Programme for International Student Assessment (PISA) 2009

Cross-cultural evidence of gender differences or similarities provides a stronger foundation for understanding the role of social and biological factors in the development of sex differences, as noted above. The aim of this study was to explore sociocultural factors that promote, or inhibit, the development of gender gaps in highly sex-typed academic domains of reading, mathematics and science [46]. It presents findings from international assessment of student abilities as part of the Programme for International Student Assessment (PISA), conducted by the Organisation for Economic Co-operation and Development (OECD). The study uses data from the most recent round of testing to calculate national and international gender gaps in reading, mathematics, and science literacy.

In addition to presenting data on national gender differences, it uses meta-analytic techniques to calculate global gender differences to examine evidence for Hyde’s *gender similarities* hypothesis [47], which posits there are no meaningful gender differences in cognitive performance. The study also seeks to replicate the findings of past researchers for the *gender stratification* hypothesis [27], [38], [43]–[44], using several measures of gender equity and occupational segregation. A number of other sociocultural constructs are also examined to determine the extent to which gender differences are culturally mediated by factors other than biology.

One hypothesised influence is the economic prosperity of a nation [39]–[41], which reflects two mechanisms. Firstly, greater economic prosperity allows for a greater proportion of national resources to be spent on education, resulting in a higher quality of education and emphasis on skills such as mathematics and science. Secondly, skills in these technical areas are in greater demand, and represent a pathway to a higher standard of living. This may result in greater competition for these occupations, and such competition may not always be helpful to the career aspirations of women wishing to enter male-dominated fields. While increases in gender equity are strongly associated with economic prosperity (and hence should be associated with smaller gender gaps), these may be partially offset by increased occupational stratification and stronger cultural stereotypes associating maths and science with gender roles [27], [32]–[33], [44]–[45]. Thus increased gender differences are not purely the result of increased spending on education and also reflect social processes.

A second mechanism by which gender differences may be culturally mediated is through the attitudes, values, and beliefs of a nation. While beliefs about the role of women in society vary considerably from nation to nation, there are few instruments available that have wide global coverage of gender stereotypes and attitudes towards women [38], [48]–[49]. One of most widely used cultural instruments is Hofestede’s [50] five cultural dimensions. One of these is theoretically relevant to cultural mediation of gender differences in cognitive ability, the dimension of power distance.

The dimension *power distance* describes the ways in which societies address the issue of human inequality, and the ways in which social groups are segregated [50]. In a lower power distance culture, there are reduced distinctions between social classes, between employees and employers, between students and teachers, and between genders. Higher power distance cultures have greater social division, and a compensatory strategy for those who are lower in power is to acquire culturally valued skills through education. Girls may have increased motivation to learn maths and science and pursue higher status occupations as a way of overcoming social inequity.

### Hypotheses

Based on prior research and theoretical perspectives, it was hypothesised that:

1. Gender differences in the domains of mathematics, and science would be found for the United States, and these would be larger than those reported by Hyde [51]. These would reflect gender stereotypes associating these domains with masculinity and males [39]. However gender differences cross-culturally would be much smaller, in partial support of a global gender similarities hypothesis.
2. Gender differences in reading performance in favor of girls would be found in reading for the United States and cross-culturally, reflecting an inherent biological disposition that is only weakly influenced by cultural environment.
3. Measures of national gender equity would be associated with smaller gender gaps in mathematics and science, in support of the gender stratification hypothesis. Furthermore, increased gender equity would be weakly associated with wider reading gaps in favor of girls.
4. Economic prosperity would be associated with wider gender gaps in mathematics and science than in less prosperous nations, reflecting increased spending on education, increased demand for these skills, and heightened competition by males. Such competition may not be helpful to the career aspirations of women, but will not influence reading performance which is less malleable to social and cultural influences.
5. Countries that score highly on Hofestede’s power distance dimension have greater segregation and foster inequalities, particularly for women. A compensatory strategy for women is to acquire culturally-valued skills such as science and mathematics. High power distance nations would be associated with smaller gender gaps or a slight female advantage in these domains. Boys may have increased motivation to develop reading and writing proficiency in high power distance cultures, resulting in smaller gender gaps for reading literacy.

## Methods

### Participants

Performance data for students accessed under PISA is offered as a publicly accessible archive for researchers. Additionally, aggregate national performance profiles are published as separate male and female subgroups [31], which were used for analysis. PISA 2009 included 34 OECD countries, as well as 31 additional partner nations. This amounts to a total participant size of 480,405 students (50.6% female) drawn from across 65 nations. This represents the most recent round of testing, as well as providing performance data for a broader range of nations than earlier PISA assessments.

### Analysis

National performance profiles in reading, mathematics and science literacy were obtained from OECD [31], which reports the assessment of boys and girls separately. Because of the large sample sizes involved in national testing, even slight or trivial differences between boys and girls may be deemed *statistically* significant, even though it may have no *practical* significance. For this reason, an effect size is presented in the form of Cohen’s *d*, the mean standardized difference. This allows the reader to draw his or her own conclusions as to the practical significance of reported gender differences.

The computation is calculated as the mean difference between male and female scores, divided by the pooled within-gender standard deviation. By convention, female scores are subtracted from male scores, so that a positive *d* indicates higher scores for males while a negative *d* reflects higher scores for females. This convention is observed for readability reasons only, and the interested reader may choose to rephrase the equations so that male scores are subtracted from female scores simply by inverting the sign of any effect size given.

Conventional criteria for labelling effect sizes as “small”, “medium”, or “large” have many limitations and should be used with great caution [52]–[53]. Cohen [53] offered a rule of thumb that an effect size of *d* ≤.20 could be considered a “small” effect for the purpose of estimating statistical power, and that many legitimate psychological phenomena studied are in fact small effects. The label of small is perhaps an unfortunate one as some researchers have mistakenly taken small to be of no practical significance, a practice Rosenthal and Rubin [54] caution against. However Hyde, et al. [20] have argued that effect sizes as small as *d* = .04 should be regarded as trivial, a cut-off which seems sound practice. Hyde [47] has also suggested that *d* ≤. 10 should be actually be regarded “as close to zero” (p.581), a cut-off which is overly conservative and dismisses what are legitimate, albeit very small, between-group differences. Accordingly, Cohen’s conventions for labelling are followed for reporting. Additionally, gender differences are presented using Rosenthal and Rubin’s [54]–[55] Binomial Effect Size Display (BESD) which presents results in a metric that represents effect size in a format suitable for interpretation by non-statisticians [56].

In order to test the gender similarities hypothesis, national gender gaps in reading, mathematics, and science were combined using meta-analysis. Comprehensive Meta Analysis (CMA) V2 software was used for the calculation of statistics [57]. A random-effects model was chosen [58] due to the high degree of cross-cultural variability, which would make a fixed-effects model unsuitable [56], [59]. Such a method is more conservative in estimating error terms and produces wider confidence intervals, giving us greater assurance that the true effect size falls within this range.

Favreau [60] argues against the use of null hypothesis testing for evaluating claims of gender difference because it may be overly sensitive, and does not present a clear picture of how differences are distributed across groups. Accordingly, data is presented showing high and low-achievers, as well as effect sizes. Even when a mean gender difference may be regarded as ‘small’ by Cohen’s [53] conventions, or ‘trivial’ by Hyde [47], a more pronounced difference may be found at the tails of a distribution in high and low-achieving students, resulting in quite disparate educational outcomes.

Moderation effects of sociocultural factors were examined to test the gender stratification hypothesis for national gender gaps using correlational analysis. Although past researchers [32], [61] have examined the gender stratification hypothesis for mathematics and reading, exploration of the relationship with science has gone largely untested. Multiple measures of gender equity were used, as each instrument operationalises the construct of gender equity differently, and prior research has shown that they vary in their predictive validity for educational and social outcomes. Other moderators tested include economic prosperity, as measured by GDP, and Hofstede’s power distance dimension.

#### Gender gap index

For comparability with Guiso, et al.’s findings, the Gender Gap Index (GGI) produced by the World Economic Forum was selected as one measure of gender equity [62]. Data for the calendar year of PISA testing was used. This measure assesses four areas: economic participation, educational attainment, political empowerment, and health and survival. While the first three are theoretical relevant to the gender stratification hypothesis, health and survival (which measures differences in male and female life expectancy, as well as sex ratio) may reflect other - largely biological – factors, thus lowering predictive validity of this measure. An additional criticism of this measure is that the economic participation component emphasises male to female participation across various sectors, but gives less emphasis to income disparities.

#### Relative status of women

As an alternative conceptualisation of gender equity, the Relative Status of Women (RSW) measures gender differences across educational attainment, life expectancy, and women’s share of income [63]. This reflects a stronger economic and educational component in estimation of gender stratification, with wage inequality playing a greater weighting.

#### Women in research

Else-Quest, et al. [61] argued that domain-specific indicators of gender equity may play an important role in the development of gender differences, with those related to gender stratification in educational outcomes showing strong predictive validity. One such marker is the relative share of research positions held by women. Data for this measure was obtained from the UNESCO Institute for Statistics, and supplemented by data from the National Science Foundation and Statistics Canada. Data was selected for the calendar year 2009 when possible, or earlier if not available. Women’s relative share of research positions was available for forty one nations.

#### Gross domestic product (GDP)

Economic data was obtained from the World Economic Outlook database produces by the International Monetary Fund. Archived information for the calendar year 2009 was obtained for sixty-one nations.

#### Hofstede’s power distance index

National power distance scores are published in Hofstede’s text *“Culture’s consequences”* [50], which ranks nations across this dimension. Data was unavailable however for many of the non-OECD partner nations, and several European countries, and was supplemented by national profiles published online (http://geert-hofstede.com). This provided coverage of fifty two nations.

### Statistical Power

While the sample size represented by the PISA 2009 was extremely large, when examining gender differences at the country level (*n* = 65) for correlation analysis the sample size is relatively small. Additionally, data for gender equity measures and for Hofstede’s cultural dimension of power distance was unavailable for many non-OECD nations reducing sample size even further. With a reduced sample size, correlations may lack sufficient power to detect relationships that are relatively weak in nature [53]. Given that hypotheses were directional (e.g. greater gender equality would be associated with a reduced gender gap in mathematics), a decision to make correlation tests *one-tailed* would often have allowed such a correlation to be deemed statistically significant (as probability values are halved). For this reason exact probability values are given, along with the size of the correlation coefficient, so that the reader can decide whether to make the appropriate adjustment. All tests report *two-tailed* correlations unless otherwise specified. Data for one nation, Colombia, represented both a univariate and multivariate outlier, and was excluded from all correlational analysis.

## Results

Although assessing qualitatively different abilities, there was a strong overlap between national gender differences in reading, mathematics and science. The quantitative abilities of mathematics and science showed the greatest overlap. Table 1 presents intercorrelations between national gender differences in these domains, while Table 2 gives correlations between national predictor variables. Tables 3 and 4 present national sample size and calculated effect sizes across the three domains for OECD and partner nations respectively.

**Table 1:** Correlations between National Gender Differences for PISA Reading, Mathematics, and Science Performance (All Nations).

	Reading	Mathematics	Science
Reading	1.00	.75***	.78***
Mathematics		1.00	.81***
Science			1.00

* *p*<.05,

** *p*<.01,

*** *p*<.001.

**Table 2:** Correlations between Measures of Gender Equity, Economic Prosperity, and Hofestede’s Power Distance Index.

	Gender Gap Index(GGI)	Relative Status ofWomen (RSW)	Relative Share of Womenin Research (WIR)	Gross Domestic Product(GDP) per capita, 2009	Hofstede’s PowerDistance Index (PDI)
GGI	1.00	.43**	−.09	.43**	−.59***
RSW		1.00	−.11	.05	−.38*
WIR			1.00	−.60***	.42**
GDP				1.00	−.58***
PDI					1.00

* *p*<.05,

** *p*<.01,

*** *p*<.001.

**Table 3:** National Gender Differences in Reading, Mathematics, and Science Literacy for Countries within the OECD.

	Sample size		Effect sizes (Cohen’s *d*)		
Country	Males	Females	Reading	Mathematics	Science
Australia	7020	7231	**−0.37**	**0.11**	**−**0.01
Austria	3252	3338	**−0.41**	**0.20**	**0.08**
Belgium	4345	4156	**−0.27**	**0.21**	**0.06**
Canada	11431	11776	**−0.38**	**0.14**	**0.05**
Chile	2870	2799	**−0.27**	**0.26**	**0.11**
Czech Republic	3115	2949	**−0.53**	**0.05**	**−**0.05
Denmark	2886	3038	**−0.34**	**0.19**	**0.13**
Estonia	2430	2297	**−0.53**	**0.11**	**−**0.01
Finland	2856	2954	**−0.64**	0.03	**−0.17**
France	2087	2211	**−0.38**	**0.16**	0.03
Germany	2545	2434	**−0.42**	**0.16**	0.05
Greece	2412	2557	**−0.50**	**0.15**	**−0.11**
Hungary	2294	2311	**−0.42**	**0.13**	0.00
Iceland	1792	1854	**−0.46**	0.04	0.02
Ireland	1973	1964	**−0.41**	**0.09**	**−**0.03
Israel	2648	3113	**−0.38**	**0.08**	**−**0.03
Italy	15696	15209	**−0.48**	**0.16**	**−**0.02
Japan	3126	2962	**−0.39**	**0.10**	**−0.12**
Korea	2590	2399	**−0.45**	0.04	**−**0.03
Luxembourg	2319	2303	**−0.38**	**0.20**	**0.07**
Mexico	18209	20041	**−0.29**	**0.17**	**0.08**
Netherlands	2348	2412	**−0.27**	**0.19**	0.04
New Zealand	2396	2247	**−0.44**	**0.08**	**−0.06**
Norway	2375	2285	**−0.52**	**0.06**	**−**0.04
Poland	2443	2474	**−0.56**	0.04	**−0.07**
Portugal	3020	3278	**−0.44**	**0.13**	**−**0.04
Slovak Republic	2238	2317	**−0.57**	0.03	**−**0.01
Slovenia	3333	2822	**−0.60**	0.01	**−0.15**
Spain	13141	12746	**−0.33**	**0.21**	**0.08**
Sweden	2311	2256	**−0.46**	**−**0.02	**−**0.04
Switzerland	6020	5790	**−0.42**	**0.20**	**0.08**
Turkey	2551	2445	**−0.52**	**0.12**	**−0.15**
United Kingdom	6062	6117	**−0.26**	**0.23**	**0.10**
United States	2687	2546	**−0.26**	**0.22**	**0.14**

Note: Significant gender differences are highlighted in bold.

**Table 4:** National Gender Differences in Reading, Mathematics, and Science Literacy for PISA Partner Countries.

	Sample size		Effect sizes (Cohen’s *d*)		
Country	Males	Females	Reading	Mathematics	Science
Albania	2321	2275	**−0.62**	**−0.12**	**−0.33**
Argentina	2183	2591	**−0.34**	**0.11**	**−0.08**
Azerbaijan	2443	2248	**−0.31**	**0.13**	**−0.10**
Brazil	9101	11026	**−0.30**	**0.19**	**0.04**
Bulgaria	2231	2276	**−0.54**	**−**0.04	**−0.19**
Colombia	3711	4210	**−0.11**	**0.43**	**0.26**
Croatia	2653	2341	**−0.58**	**0.12**	**−0.10**
Dubai (UAE)	5554	5313	**−0.47**	0.02	**−0.26**
Hong Kong-China	2257	2280	**−0.39**	**0.15**	0.03
Indonesia	2534	2602	**−0.55**	**−**0.02	**−0.13**
Jordan	3120	3366	**−0.63**	**−**0.01	**−0.39**
Kazakhstan	2723	2689	**−0.47**	**−**0.01	**−0.10**
Kyrgyzstan	2381	2605	**−0.54**	**−0.07**	**−0.24**
Latvia	2175	2327	**−0.59**	0.02	**−0.09**
Liechtenstein*	181	148	**−0.39**	**0.28**	0.18
Lithuania	2287	2241	**−0.68**	**−0.07**	**−0.20**
Macao-China	3011	2941	**−0.45**	**0.13**	**−**0.03
Montenegro	2443	2382	**−0.57**	**0.14**	**−0.14**
Panama	1936	2033	**−0.33**	**0.06**	**−**0.02
Peru	3000	2985	**−0.23**	**0.20**	**0.05**
Qatar	4510	4568	**−0.44**	**−0.05**	**−0.25**
Romania	2378	2398	**−0.47**	0.04	**−0.13**
Russian Federation	2623	2685	**−0.50**	0.03	**−**0.03
Serbia	2680	2843	**−0.47**	**0.13**	**−**0.01
Shanghai-China	2528	2587	**−0.50**	**−**0.01	**−**0.01
Singapore	2626	2657	**−0.32**	**0.05**	**−**0.01
Chinese Taipei	2911	2920	**−0.43**	**0.05**	**−**0.01
Thailand	2681	3544	**−0.52**	**0.05**	**−0.16**
Trinidad and Tobago	2283	2495	**−0.51**	**−0.08**	**−0.17**
Tunisia	2359	2596	**−0.37**	**0.16**	0.01
Uruguay	2810	3147	**−0.42**	**0.13**	**−**0.01

Note: Significant gender differences are highlighted in bold.

* Although effect sizes are large, caution must be taken interpreting due to small sample size.

### Reading Literacy

Table 5 presents summary statistics for reading achievement. Within the United States, girls outperformed boys in overall reading, Cohen’s *d* = −.26 which is just over a quarter of a standard deviation. By comparison, the OECD gender difference in reading was larger, *d* = −.42. Examining performance data for the US sample further, boys were overrepresented at the lowest level of reading proficiency, with approximately 4.5 boys to every girl. When we consider the vocational and economic outcomes associated with poor literacy, such a large disparity is alarming. Such findings are consistent with previous findings on reading literacy assessed by PISA [32] and gender differences in the prevalence of reading difficulties [64]–[65]. When we look at students attaining the highest level of reading proficiency (Level *6*), the trend is reversed with over twice the number of girls than boys achieving the highest standard. Thus boys are overrepresented at the lower end of the spectrum, while girls are overrepresented at the highest end.

**Table 5:** Reading Ability for Girls and Boys for the USA and OECD nations.

	Girls	Boys	Standard Deviation	Effect Size (*d*)
United States	513	488	(97)	−.26
OECD Average	513	474	(93)	−.42
*% students at lowest ability level, USA*	0.2%	0.9%	4.5 boys : 1 girl	
*% at highest ability level, USA*	2.1%	0.9%	2.4 girls : 1 boy	

Overall, across all sixty-five nations the gender difference in reading literacy favored girls, *d = *−.44 [95%CI = −.41, −.46], *Zma* = –31.04, *p*<.001, with a similar gender difference also being found for OECD nations only as a group. Additionally, statistically significant gender differences in reading favoring girls were found in *every* nation surveyed, and have since the first assessment in 2000 [66]. These effect sizes ranged from −.11 to −.68, from a small- to a medium- sized difference in reading literacy.

To investigate the gender stratification hypothesis, I examined correlations between gender equity and the gender gap in reading. Partial support was found for the gender stratification hypothesis. National scores on the Relative Status of Women (RSW) measure were negatively correlated with reading, *r* = −.33, *p* = .018, such that increased gender equality was associated with larger reading gaps favoring females. Additionally, the educational measure of women in research (WIR) was associated with larger reading gaps, *r = *−.38, *p* = .016. Surprisingly though, there was no association between the gender gap index (GGI) and reading ability, *r = *01. Examination of the scatterplot showed no discernable pattern, and the result was not driven by outliers.

Stronger support for the gender stratification hypothesis was found when examining gender differences in the percentage of students attaining the highest level of reading. Improvements in national gender equity was associated with a wider gender gap in high achieving girls, RSW, *r* = −.32, *p* = .021; GGI, *r* = −.41, *p* = .002, which is consistent with the findings of Guiso, et al. [32]. Somewhat surprisingly, however, the educational measure of gender equity showed a strong positive association, with increases in the percentage of women in research associated with smaller gender gaps, *r* = .57, *p*<.001. While the role of women in higher education may make a contribution to the mean performance of girls and boys in *basic* reading literacy, it may be the case that for high-achieving reading comprehension skills, boys and girls benefit equally from female role-models in higher learning.

No association between GDP and gender differences in reading was found, *r* = .04, consistent with predictions. However, a strong association with economic prosperity was found for reading high achievers, *r* = −.43, *p*<.001 with a greater ratio of female to male high achievers as GDP increased. This suggests an interaction between gender and GDP, with girls benefiting more from economic prosperity than boys. Furthermore, while no association was found between power distance and mean reading literacy scores of boys and girls, a strong positive association with the gender gap in high achievers was found as hypothesized, *r* = .40, *p* = .003 with gender ratios approaching more equal representation as power distance increased. Cultural mediation through economic prosperity and power distance was not found for mean male and female performance, only for gender ratios in high achievement.

### Mathematics Literacy

Table 6 presents summary statistics for mathematics literacy. Within the United States, boys scored higher on mathematical literacy than girls, *d = *.22 which is a small but non-trivial effect size. Additionally, the size of the gender differences was almost twice that of the OECD average. This is in contrast to previous studies examining national mathematics performance by Hyde, et al. [20] which had found a gender gap that approached zero. At the lower end of ability level for the US sample, the difference in prevalence between girls and boys was extremely slight; however at the highest ability levels there were just over twice as many boys than girls reaching this proficiency level.

**Table 6:** Mean Mathematical Ability for Girls and Boys for the USA and OECD nations.

	Girls	Boys	Standard Deviation	Effect Size (*d*)
United States	477	497	(91)	.22
OECD Average	490	501	(92)	.12
*% students at lowest ability level, USA*	9.5%	6.8%	1.40 girls : 1 boy	
*% at highest ability level, USA*	1.2%	2.5%	2.12 boys : 1 girl	

As the distribution of gender differences differed somewhat between OECD and partner nations, they are reported separately. Overall, across all 34 OECD nations, there was a significant gender difference favoring males on mathematical literacy, Cohen’s *d* = .13 95%CI [.11,.15], *Zma* = 11.22, *p*<.001. While this is a small effect size, it does exceed the criteria set forth by Hyde and Linn [51] for trivial gender differences. Gender differences across PISA partner nations also favored males, Cohen’s *d* = .07 95%CI [.02,.11], *Zma* = 3.10, *p* = .001 although this difference was somewhat smaller.

**Figure 2:**
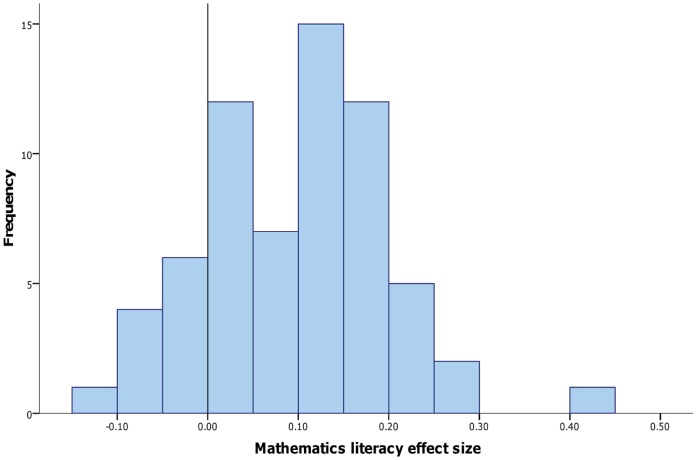
Histogram of gender difference effect sizes in mathematics literacy across OECD nations.

While statistically significant differences were found in most countries, they showed considerable variability ranging from *d* = −.12 to *d* = .43 (see Figure 2). For many nations the gender gap is negligible, while others show small to medium sized differences. Additionally the direction of the gender gap was sometimes reversed, with girls outperforming boys in many nations. Under different social and educational environments, a gender advantage supporting either males or females emerges. This would be inconsistent with Hyde’s [47] *gender similarities* hypothesis; rather, gender differences or similarities in mathematics are strongly mediated by cultural factors.

To explore the *gender stratification* hypothesis, correlations between gender equity measures and the gender gap in maths were examined. As hypothesized there was a strong negative relationship between the educational measure of women in research and the gender gap in mathematics, *r* = −.38, *p* = .014. Greater representation of women in research was associated with smaller gender gaps or a female advantage, consistent with the findings of Else-Quest, et al. [61]. However, only a weak association was found between gender equity measure of RSW, *r* = −.14, and no association was found between GGI and maths, in contrast to the findings of Guiso, et al. [32].

Since the PISA 2009 dataset includes a much broader range of partner nations than was examined by Guiso, et al. [32], the strength of the gender equity association may have been obscured by additional noise reflecting developed/developing nationhood. When restricting analysis to OECD nations only, the hypothesized gender equity association was found for the relative status of women (RSW) measure, *r* = −.42, *p* = .020, as well as a weak association with GGI, *r* = −.21 that fell short of statistical significance. While gender equity plays an important role in the development of gender differences in mathematical literacy for developed nations, it may be the case that there are more proximate needs for girls in developing nations (such as access to schooling, parental support, freedom from work and home duties) that these gender equity measures do not assess.

**Figure 3:**
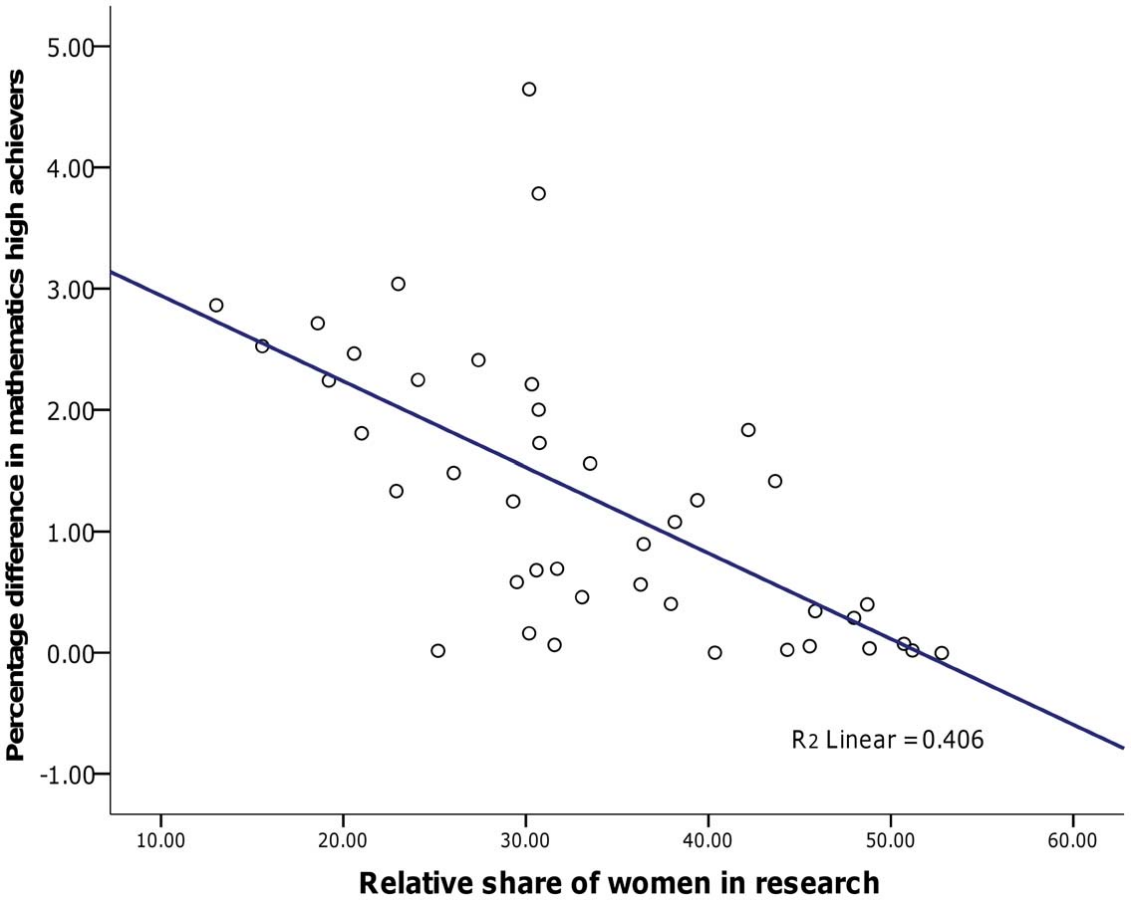
Relationship between women in research and gender ratios of high-achievers in mathematics literacy.

A similar pattern of associations was found for gender differences in high achieving mathematics students across all nations. There was a strong association between women in research educational measure, *r* = −.63, *p*<.001, with increased representation of women in research positions associated with a smaller gender difference in high achievers approaching zero (see Figure 3). However no association was found between the gender gap in high achievers and other gender equity measures, nor was this found when restricting to OECD nations only.

Support was also found for the economic prosperity hypothesis. Mean gender differences in mathematics literacy were larger in more economically prosperous nations, *r* = .31, *p* = .015. This relationship was stronger for high achievement, *r* = .53, *p*<.001 with a greater number of males attaining this level of proficiency.

Examining the relationship between Hofsetede’s power distance cultural dimension and mathematics literacy, support was also found for cultural mediation. There was a strong negative relationship between power distance and mean gender differences in mathematics, *r* = −.28, *p = *.044, as well as for gender ratios in high achievement, *r* = .−33, *p* = .019. Gender differences were smaller in nations with greater tolerance for inequality, suggesting a compensatory strategy to acquire culturally and economically valued skills in mathematics.

### Science Literacy

**Table 7:** US National Science performance for girls and boys, including high and low achievers.

	Girls	Boys	Standard Deviation	Effect Size (*d*)
United States	495	509	(98)	.14
OECD Average	501	501	(94)	.00
*% students at lowest ability level, USA*	4.6%	3.8%	1.20 girls : 1 boy	
*% at highest ability level, USA*	1.0%	1.5%	1.52 boys : 1 girl	

Table 7 presents summary statistics for science literacy achievement scores. For the United States, a gender difference of *d = *.14 was found. Furthermore, the United States showed the largest gender difference across *all* OECD countries. Although statistically significant, the difference between the average boy and girl is small, but neither is it of a trivial magnitude either. Boys in the US scored higher than boys internationally, while girls scored lower than their international peers. Additionally, at both ends of the ability level spectrum, gender differences were more pronounced – there are approximately 1.5 boys to every girl achieving the highest level of science proficiency. Thus while the mean difference between males and females may be “small” by Cohen’s [53] effect size conventions, it may have more of an impact than one might assume from that label.

**Figure 4:**
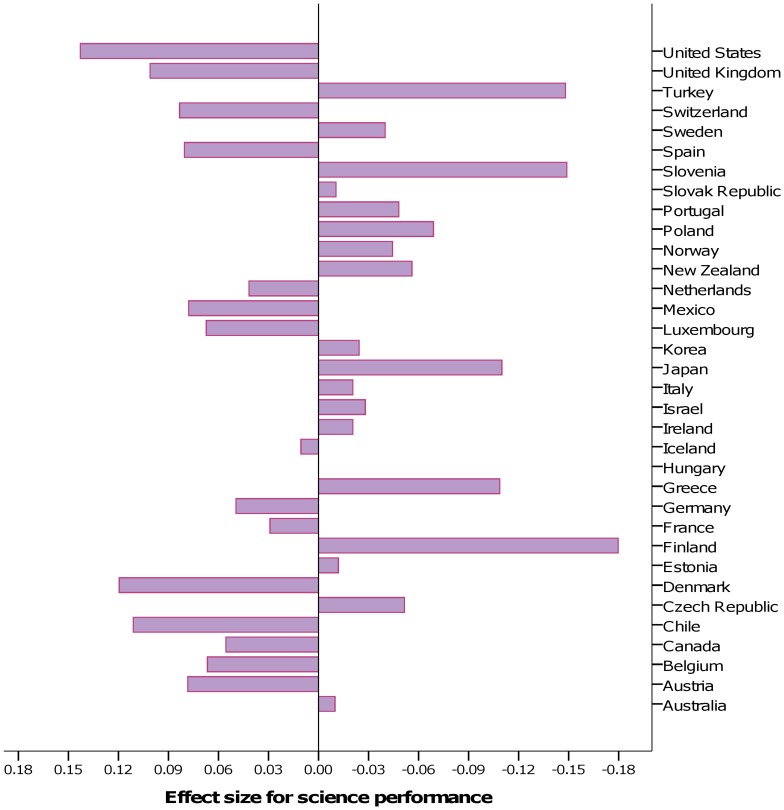
Distribution of effect sizes for gender differences in science literacy across OECD nations.

In contrast to US performance, across OECD countries there was no difference between boys and girls, *d* = .00 95%CI [−.03,.03], *Zma* = 0.10, *p* = .919. However there was a large degree of cultural variability, with gender differences favoring both boys and girls. Indeed, statistically significant differences in favor of boys were only found in nine countries, and only three were higher than the ‘close-to-zero’ criterion suggested by Hyde of *d* <.10. Figure 4 shows mean standardized effect sizes. (Cohen’s *d*) for gender-gaps in science across OECD nations. Gender similarities, rather than differences, were the norm which is consistent with the findings of Hyde and Linn [51]. Somewhat surprisingly, there were also five nations where girls *outperformed* boys to a statistically significant degree (the largest being Finland, *d* = −.17). One of the advantages of cross-cultural comparisons in national testing is that it highlights just how powerfully cultural and environmental influences can be in either promoting - or inhibiting - the cognitive development and learning of a child.

A markedly different picture of gender differences in science can be found across the 31 non-OECD nations. In general, females scored higher in science literacy than males across most nations. Overall, across non-OECD nations surveyed there was a statistically significant difference in science literacy favoring girls, *d* = −.09 95%CI [−.14, −.04], *Zma* = −3.44, *p* = .001. For some nations, the gender difference was trivial or favored boys, but these were the exception; this is in contrast to the gender similarities in science noted above for OECD nations.

When both OECD and non-OECD nations were combined, there was a statistically significant difference in favor of girls, *d* = −.04 [95%CI−.070,−.013] *Zma* = −2.84, *p* = .005. This effect size would fall into the trivial size by Hyde’s [67] conventions, but a focus on the combined sample overlooks the pattern of gender differences at a national level where girls show small but meaningful gains over boys in science literacy across large parts of the world. Given that women are underrepresented in science, particularly in the United States [68] such findings call into question the validity of cultural stereotypes that associate science with masculinity [69], and highlight the need for further efforts at challenging these damaging cultural stereotypes.

Examining mean gender differences in science literacy, partial support for the gender stratification hypothesis was found. There was a strong correlation between national GGI scores and science, *r* = .29, *p* = .035, with greater gender equity associated with smaller gender gaps approaching zero. However, only a weak non-significant association was found for the RSW, *r = *.14.

Additionally, there was a strong negative correlation between the percentage of women in research and gender gaps in science, *r* = −.39, *p = *.011, with increased representation of women being associated with a stronger female advantage over males in science. Thus increased gender equity was associated with more equal science performance, but this was offset by higher female performance as the share of women in research positions increased.

**Figure 5:**
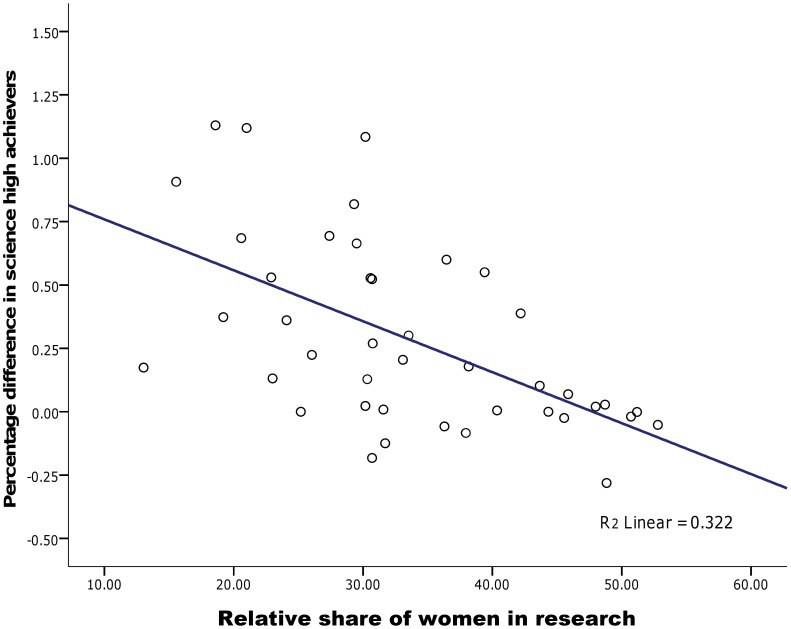
Relationship between women in research and gender ratios of high-achievers in science literacy.

Only weak support for the gender stratification hypothesis was found for gender differences in high achievement in science. Increased gender equity as measured by the percentage of researchers who are women was associated with smaller gender gaps in the number of high achievers, *r* = −.57, *p*<.001 (see Figure 5). While positive female role models are certainly important for challenging gender stereotypes about women in science generally, they may be even more so for encouraging young women to excel in science and pursue it as a career path. In contrast to this finding, there was no association between the relative status of women measure, *r* = .12 and a slight positive correlation with gender equity as measured by the GGI, *r* = .29, *p* = .029, with increased gender equity associated with more male high achievers than female which is contrary to predictions. This anomalous association may be at least partly explained by the underlying construct measured by the GGI. It incorporates a strong economic component in its formula, with a correlation of *r* = .43 between national GGI scores and economic productivity as measured by GDP. When controlling for economic productivity, the association between GGI and science high achievers becomes non-significant, *r* = .12, *p* = .373.

Strong support was also found for culturally mediation of gender differences in science. Positive relationships were observed between GDP and gender differences in mean science scores, *r* = .42, *p* = .001, as well as for gender ratios in high achievement, *r* = .27, *p = *.036, as hypothesised. In contrast, a negative relationship was found between the power-distance dimension and mean gender differences in science, *r* = −.39, *p* = .005 with gender differences favoring girls in high power-distance nations. This effect was even stronger for gender ratios in high achievement, *r* = −.45, *p* = .001.

## Discussion

Does the size of gender differences in reading, mathematics, and science from PISA assessment merit further research into the social and cultural factors that promote, or inhibit, differential educational outcomes for boys and girls? Evidence presented for the United States shows that there are meaningful gender gaps across all three domains. Furthermore, they are larger than those found in most OECD nations placing the US among the highest gender gaps in mathematics and science in the developed world, but somewhat smaller than other nations in reading literacy. However, quite different patterns are found when examining gender gaps globally. US performance is reviewed first, followed by a discussion of cross-cultural evidence.

### Reading Literacy

While a small-to-medium sized gender difference in reading was found for US students *d* = −.26, this was comparatively smaller than that found in other OECD nations. However, gender differences were strikingly different at both tails of the distribution, with boys overrepresented in the lowest level of reading proficiency and girls overrepresented in the highest. PISA sampling allows for exclusion of students with limited language proficiency, so it is likely that this result reflects poorer reading ability generally rather than male overrepresentation in reading difficulties students. This pattern is consistent with existing research on gender ratios for reading difficulties [64]–[65].

Cross-culturally, a medium sized gender difference (*d = *−.44) was found for reading literacy, which would be inconsistent with Hyde’s gender similarities hypothesis [47]. Expressed in the BESD format, the likelihood of being average or higher in reading ability increases from 39% for boys to 61% for girls. Reading performance was higher for girls than boys across *every* nation, but also showed considerable between-nation variation. Though the direction of gender differences would be consistent with a biological explanation, it appears at least partially malleable by social and cultural factors. While there was no support for cultural mediation through economic prosperity and power distance in mean gender differences, contrary to predictions associations were found for high achievers in reading literacy.

It has been a common research finding that boys are generally poorer readers and writers than girls [70], and considerable effort has been made to address the gender gap over recent decades with focus on early identification and intervention for reading difficulties. Basic literacy is an essential life skill for all children, and for full participation as a citizen. While much attention is given to the issue of math and science gender gaps, gender gaps in reading are in fact much larger and favor girls at both tails of the distribution. While gender gaps in reading literacy for the USA were smaller than those found internationally, the need for further progress remains. Enrolments of women outnumber men in college, with higher female GPA and completion rates than their male peers [16], [65]–[66]. Raising the educational aspirations of boys who experience difficulties in reading literacy, and continuing support for early intervention is critical as a matter of gender equity.

### Mathematics Literacy

Gender differences in mathematics literacy were comparatively larger for the United States than those found across other OECD nations. These findings are consistent with student test data reported by Hedges and Nowell [23], as well as findings from PISA 2003 [32], [61] that a small gender difference in mathematics exists, but is also inconsistent with findings of no difference reported by Hyde and colleagues using data from the NAEP [20]. How are we to reconcile this discrepancy?

As reviewed earlier, problem-solving for complex and novel mathematics tasks show a small to medium sized male advantage [30], and PISA assessment of mathematical literacy is somewhat different to that of the NAEP. This may allow for greater differentiation between high and low ability students if a ceiling-effect is present, and may provide a more thorough test of the gender similarities hypothesis. It may well be the case that gender differences in basic mathematical literacy are trivial in size [71], but that gender differences can be found in more complex tasks [30] requiring more than just curriculum knowledge.

Gender differences were observed for US performance, *d = *.22, which is small in size by Cohen’s [53] conventions and non-trivial by Hyde’s [47] criteria. When expressed in the BESD format, the likelihood of being average or higher in mathematics increases from 44.5% for girls to 55.5% for boys. One should be careful not to make too much, or too little, of this gender difference. As Hyde [47] points out, the degree of overlap between male and female performance is large for effect sizes in the small range, with many girls performing at or above the male average in mathematics. This perspective does not diminish the observation that a gender gap exists. As can be seen from the cross-cultural evaluation of mathematics, gender gaps in mathematics are not an inevitability, with many countries in fact showing higher female performance.

This difference is most apparent when examining student attainment of the highest proficiency level in mathematics, with double the amount of boys than girls reaching this stage. Benbow [22] argued that gender differences in high-achievement for mathematics could be at least partially explained by greater male variability and a combination of biological and environmental factors. It is likely that greater male variability explains at least part of the gender difference in high achievement, but that sociocultural factors also play a role in the development of mathematics at the extreme tails of the distribution. While general proficiency in mathematics is an important life goal for *all* students, attainment of an advanced level of mathematics is an important prerequisite for pursuing more technical degrees in STEM-related fields [72]. A growing body of research suggests that self-efficacy and confidence in mathematics play an important part in the decision making process of women to pursue STEM-related careers or direct their talents elsewhere [23], [62]–[64]. Increasing self-confidence in mathematics and instilling a sense of mastery may be a crucial component any educational intervention, as well as challenging negative cultural stereotypes about women’s ability in mathematics [41], [69]. At least for students within the USA, gender differences in mean and high achievement for mathematics have not been eliminated, and highlight the need for further progress.

While cross-culturally, gender differences favored males across OECD and partner nations, the magnitude of this difference (*d = *.13) was also small in size and subject to wide cultural variation. The likelihood of being average or higher in mathematical ability increases from 46.7% for girls to 53.2% for boys, a small but non-trivial difference. Unlike reading literacy, there were a number of countries which had non-significant gender differences, which would be inconsistent with strong biological differences between boys and girls in mathematical reasoning [11], [15], [65]–[66]. It may be the case that whatever slight advantage boys have is magnified by social and cultural reinforcement, to produce gender differences in some countries but that other nations raise girls and boys to equivalent performance.

A parallel may be also drawn between cross-cultural support for gender differences in mathematics, and similar evidence for gender differences in spatial ability [67]–[70]. Many theorists have argued that spatial ability provides a foundation for later development of mathematical ability [13], [73]–[76]. Although gender differences are consistently found across *all* cultures favoring males, the magnitude of spatial differences is subject to cultural variation. In particular, Lippa, Collaer and Peters [77] compared national measures of gender equality and economic development with gender differences in spatial performance for a fifty-three nation sample, finding strong positive correlations with both measures. These findings are correlational, not causal, but taken together may change the way in which we think about the development of cognitive differences. It would appear that gender differences in number of cognitive abilities are at least partially influenced by social and cultural influences such as gender equality and the status of women [32], [61]. While parental, teacher and peer influences also play a part [78]–[83], the influence of wider cultural influences at the macro-level may be important considerations for any biopsychosocial models of gender difference.

### Science Literacy

While the effect size for gender differences in science literacy for the USA was relatively small compared to that of reading and mathematics, it stands out as the largest effect size across all OECD nations, *d* = .14. This is a small effect size, but also not a trivial one by Hyde’s [47] conventions. Represented in the BESD format, the likelihood of being average or higher in science literacy increases from 46.5% for girls to 53.5% for boys. Additionally, boys were slightly overrepresented in attaining the highest level of science proficiency, but not to the same degree as for mathematics. Of all the domains assessed, science literacy appears to be the most variable cross-culturally, with many countries showing no difference whatsoever, and many showing a female advantage. This is a promising sign, and a benchmark to which the USA can aspire. This pattern of results was consistent with the gender similarities hypothesis.

### Gender Stratification Hypothesis

In order to test the gender stratification hypothesis, this study examined the relationship between national measures of gender equity and gender gaps in reading, mathematics and science literacy. While some support for the gender stratification hypothesis was found, the predictive validity of gender equity measures varied across instruments and domains. In particular, relationships between the Gender Gap Index instrument were often weak, and in the case of science literacy high achievers in a direction contrary to hypotheses. This failure to support the gender stratification hypothesis using all gender equity measures should not be interpreted as a refutation of the hypothesis, but means that one should evaluate the hypothesis carefully. Each instrument taps different aspects of the underlying gender equity construct, and it is likely that some elements of equity have greater bearing on educational outcomes than others. A consistent finding across all three domains, and across both mean performance and high achievers, was that the relative share of women in research accurately predicted the presence or absence of gender differences. However, composite measures of gender equity showed weaker or inconsistent associations.

It may be the case that measures more closely related to education, such as gender differences in relative share of research and science positions, may more accurately measure the underlying social and cultural conditions that foster or inhibit the development of gender differences in reading, mathematics and science literacy. None of the instruments directly measure attitudes towards women in STEM-related fields, or gender stereotypes about the relative abilities of males and females [69], [84]. Instead, the composite measures relate to the role of women in society in general, which may lack the specificity required to consistently predict gender differences in learning outcomes. Although increased gender equity generally may be associated with the presence or absence of gender gaps in reading, mathematics and science, it may not be the direct cause.

The relative share of women employed in scientific research may be more directly related to societal attitudes about the role of women in technical fields, and to gender stereotypes about the capabilities of males and females in sex-typed achievement domains. Girls growing up in a society that praises the scientific and technical achievements of men but lacks equivalent female role models may perceive that women are less capable in this area, or that their skills are not culturally valued. They may instead be motivated to develop other talents, such as high proficiency in language, and to pursue careers in less-segregated professions. Conversely, if girls grow up in a social environment where they see progression into further education and specialisation in STEM-related fields is not only possible but also commonplace, they may be more motivated to acquire and master mathematics and science skills. In such a culture, encouragement from parents and teachers may be higher, and they may show greater confidence and improved self-efficacy in these domains than children from other cultures. While mean gender differences are smaller (or favor females) in such nations, this also translates to increased female representation in high achievers as well. This provides for stronger support of the gender stratification hypothesis.

### Economic Prosperity

Mean gender differences were larger for mathematics and science in economically prosperous nations as hypothesised but were largely unrelated to reading literacy. This likely reflects both increased educational spending for economically prosperous nations, as well as increased emphasis being placed on mathematics and science skills. Student achievement in less prosperous nations may be more homogenous with smaller gender differences, and there may be a reduced focus on teaching of these skills. It may also be the case that there is greater competition by males to achieve in these masculine sex-typed domains. These associations were also found for gender ratios in high achievement. Additionally, gender ratios for high achievers in reading literacy were also related to economic prosperity, which was unexpected.

### Power Distance

Hofstede [50] argued that cultures differed in their tolerance for inequality, with some cultures observing social class distinctions more strongly than others. Such cultures may place greater emphasis on social roles and stratification, but one way of overcoming inequity is the pursuit of culturally valued skills and traits. As a compensatory strategy, girls may seek out higher social status positions by obtaining education in mathematics and science, and this may help to explain the female advantage for science observed for non-OECD nations. As hypothesised, these associations were found for mean gender differences in mathematics and science as well as for gender ratios of high achievers. Lesser support was found for cultural mediation in reading literacy, with no association for mean gender differences but a positive association for gender ratios in high achievement.

### Social Implications

The question of whether gender differences exist in cognitive abilities has important implications for parents, educators, and policy-makers [20], [47], [72], [82]–[83]. Yet great caution must be taken when interpreting empirical evidence - Hyde [47] raises a legitimate concern that inflated claims of wide gender difference might contribute to increased gender segregation in education and the workforce, and that the potential of girls may be overlooked by parents and teachers [78]–[82]. This study finds evidence of gender similarities rather than differences cross-culturally but also that meaningful gender gaps in maths and science remain and are related to cultural factors.

Society as a whole also has a vested interest in this question, both directly and indirectly. We as citizens rely on the services and advancements that a highly skilled science and technology workforce provide, with direct benefits for our health and lifestyle, and for an economy that depends on the brightest and most innovative of minds entering these fields to sustain an internationally competitive advantage. There are also indirect benefits from having a society that is at least partially scientifically literate – making decisions through the political process and personal choices about issues such as the use of stem-cell technologies, vaccination of children against disease, or evidence of climate change. When students, particularly girls, disengage with science learning there are costs to the individual, in the form of reduced security and income, but also to the wider society. While not every child may have the ability or interest to pursue a scientific career, a basic scientific literacy is required for full participation in society.

The underrepresentation of women in science is a serious social issue, and considerable resources are being expended to address this problem [72], [83]–[84]. Recognising that a gender gap exists is the first step towards changing it, while cross-cultural evidence of gender similarities provides strong evidence that the gender gaps in maths and science are not inevitable. STEM-related careers can be a pathway to a higher standard of living and job security, and girls deserve the same encouragement as boys to pursue these professions as a matter of social justice. Newcombe et al. [85] argues that psychology can make a positive contribution to changing the social and educational environments that curtail the potential of all students in mathematics and science.

### Strengths and Limitations

The broader coverage of nations included in the PISA 2009 round of assessment makes for a stronger test of research hypotheses than was previously possible. Additionally, many of the partner nations would be categorised as lower in human development, with reduced access to the educational advantages found in other nations. While researching educational outcomes for large and economically prosperous nations like the United States is important, debate about gender differences is often shaped by evidence from relatively affluent samples. In less advantaged nations, provided girls and boys are still afforded the same access to education, performance in maths and science literacy is more homogenous giving greater support to the gender similarities hypothesis. However, there is still substantial cultural variability in gender differences, and much of this is driven by cultural variation in gender equality. For a large portion of the world, the strongest predictor of gender differences in educational outcomes is equivalent access to education, occupational segregation, and representation of women in technical and research professions. If priority were to be given to improving these globally, substantial improvements in female literacy in maths and science could be realised.

While support for research hypotheses were generally observed, availability of data for cross-cultural correlations meant reduced statistical power to detect relatively weak correlations. It may well be the case that the hypothesised associations with mean gender differences across reading, maths, and science could have been detected with expanded coverage of Hofstede’s cultural dimensions [50]. There are likely many other cross-cultural correlates of gender differences that remain unexplored, such as gender stereotypes about cognitive abilities, and cultural variations in attitudes towards women in society. Such research is limited by the need to obtain wide coverage of these constructs across nations.

### Summary

Evidence from national testing for the United States shows that there are meaningful gender gaps to be addressed in academic achievement across reading, mathematical and science literacy. Furthermore, these are larger than that found cross-culturally, where evidence for the gender similarities hypothesis is stronger. Globally, there is a small gender difference in mathematics literacy favoring males, and a small difference in science literacy favoring girls in non-OECD nations. However, a consistent finding for reading literacy is that girls outperform boys both in mean differences overall and gender ratios in attaining high reading achievement. Correlational analyses show that economic prosperity, gender equity, and the dimension of power distance are good predictors of global gender differences in cognitive abilities.
